# A multi-stakeholder evaluation of the Baltimore City virtual supermarket program

**DOI:** 10.1186/s12889-017-4864-9

**Published:** 2017-10-23

**Authors:** Pooja Lagisetty, Laura Flamm, Summer Rak, Jessica Landgraf, Michele Heisler, Jane Forman

**Affiliations:** 1 Division of General Internal Medicine at University of Michigan, Ann Arbor, MI USA; 2 VA Ann Arbor Center for Clinical Management Research, North Campus Research Center, University of Michigan, 2800 Plymouth Road, Bldg 16 Rm 345E, Ann Arbor, MI 48109 USA; 3 Institute for Health Policy and Innovation, University of Michigan, Ann Arbor, MI USA; 4 Baltimore City Health Department, Baltimore, USA; 5 Michigan State University, East Lansing, MI USA

**Keywords:** Food access, Technology, Underserved populations

## Abstract

**Background:**

Increasing access to healthy foods and beverages in disadvantaged communities is a public health priority due to alarmingly high rates of obesity. The Virtual Supermarket Program (VSP) is a Baltimore City Health Department program that uses online grocery ordering to deliver food to low-income neighborhoods. This study evaluates stakeholder preferences and barriers of program implementation.

**Methods:**

This study assessed the feasibility, sustainability and efficacy of the VSP by surveying 93 customers and interviewing 14 programmatic stakeholders who had recently used the VSP or been involved with program design and implementation.

**Results:**

We identified the following themes: The VSP addressed transportation barriers and food availability. The VSP impacted customers and the city by including improving food purchasing behavior, creating a food justice “brand for the city”, and fostering a sense of community. Customers appreciated using Supplemental Nutrition Assistance Program (SNAP) benefits to pay for groceries, but policy changes are needed allow online processing of SNAP benefits.

**Conclusions:**

This evaluation summarizes lessons learned and serves as a guide to other public health leaders interested in developing similar programs. Provisions in the U.S. Department of Agriculture (USDA) Farm Bill 2014 allow for select grocers to pilot online transactions with SNAP benefits. If these pilots are efficacious, the VSP model could be easily disseminated.

## Background

Roughly two-thirds of US adults are overweight and obese. [1] With obesity rates much higher than previous generations and healthcare costs of nearly 200 billion dollars related to obesity [2], public health agencies have placed emphasis on developing strategies to prevent obesity. [1] One such strategy has emphasized increasing access to supermarkets and grocery stores that stock healthy foods such as fruits and vegetables.

Poor access to supermarkets and grocery stores has been associated with increased rates of obesity [3]. However, these findings are often equivocal based upon what population is studied and how access to supermarket is defined. [4, 5] Obesogenic environments, which include increased access to fast food restaurants and decreased access to grocery stores and supermarkets, disproportionally affects rural, minority and low-income populations [6, 7]. Data from Baltimore City has shown that African-American, lower-income neighborhoods have substantially lower access to healthy foods compared to white, high-income neighborhoods [8] and suffer from a 20-year life discrepancy compared to higher-income neighborhoods due to nutrition-related illnesses such as cardiovascular disease. [7, 9]

In response to this evidence, multiple public health agencies including the Baltimore City Health Department (BCHD) have developed policy initiatives aimed to reduce obesity by providing residents with healthier food options through expanding access to supermarkets [10, 7]. However, building brick and mortar supermarkets in disadvantaged neighborhoods is often costly and not feasible. Developing online supermarkets delivery options for low-income populations is a potential solution to brick and mortar supermarkets that has been underexplored and evaluated.

In 2010, the Baltimore City Health Department (BCHD), launched the Virtual Supermarket Program (VSP), a large-scale online food delivery pilot. Through city-wide collaboration with the Enoch Pratt Free Library system, local public schools, local grocers and local universities, the VSP is the first online grocery store pilot in a major US city that seeks to provide a cost-effective solution to increasing access to healthy foods in food deserts, defined as an area where the distance to a supermarket is more than 0.25 miles, the median household income is at or below 185% of the federal poverty level, vehicle ownership is low, and the average Healthy Food Availability Index score, which measures the presence of eight essential food groups (e.g. milk, fresh fruits, and fresh vegetables) in a region for all food stores is low [11].

In this study, we employed mixed quantitative and qualitative methods to evaluate the perspectives of multiple stakeholders, including both customers and program partner representatives. We aimed to identify key barriers to food access among low-income Baltimore City neighborhoods and whether and if the VSP addresses those barriers. We also explored more broadly how to effectively build partnerships and overcome policy barriers to create a sustainable program.

## Methods

### Intervention

The VSP pilot uses an online grocery ordering system to bring healthy food options to neighborhoods not served by grocery stores and supermarkets. Participants using the program go to a hub within walking distance such as a library, school, or senior housing center and place their grocery orders online with the assistance of the BCHD. The grocer aggregates and delivers the groceries to the hub the following day within two-hour time windows to insure freshness of food where customers can pay with cash, credit, or Supplemental Nutrition Assistance Program (SNAP) benefits (as long as customers are physically present at grocery pick-up times). Grocery orders can be placed from any computer but ordering assistance is provided to those needing it at the hubs. With current SNAP regulations, customers must use those benefits via an electronic banking card at the point of food pick up and cannot enter this information at the time of ordering. BCHD first implemented the program in March of 2010 at local neighborhood library branches. It has since expanded to include schools, senior living facilities, and public housing facilities. From March 2010 to July 2016, the program has facilitated over 8200 grocery orders for over 900 unique customers across 16 community sites. In the July 1, 2015 to June 30, 2016 fiscal year alone, the program served 403 unique customers across 11 community sites, who placed 3640 orders, averaging $29.85 per order. Until July 2015, the program was funded through foundation grants.

BCHD staff provides computers, manual assistance to place orders, and subsidies to grocers to offset delivery costs. Aggregating the delivery reduces delivery costs allowing the grocer to deliver food to a safe, low-crime location. In addition, BCHD offers food coupons to customers to incentivize healthy food purchases.

Local art students partnered with BCHD to create a marketing campaign to promote the VSP. The students focused on user-centered feedback from customers to design bus ads, bookmarks, grocery bags, flyers, visually appealing coupons, and posters to advertise about the program. In 2011, the program also took on a community-based partnership approach and employed Neighborhood Food Advocates (NFA), defined as community members who serve as liaisons between the BCHD and the neighborhood by taking on multiple roles including recruiting customers, organizing orders and deliveries, and helping customers place orders. In 2013, the program was suspended for less than one year as the initial participating supermarket closed and a new supermarket was secured. The program restarted in 2014 and has been running continuously since then. It currently receives funding from private and foundation grants and the City of Baltimore.

### Setting

As of 2014, Baltimore City had a total population of 622,793 people, of whom 63.1% are African –American and 28.2% Non-Hispanic whites [12]. At the program’s inception, all neighborhoods participating in the VSP were classified as food deserts. In recent years, BCHD has expanded program eligibility to include low-income apartment buildings located more than 0.25 miles from a grocery store, regardless of area income or vehicle ownership. Since 2010, the following 11 Baltimore City neighborhoods have been serviced by 16 VSP sites: Bolton Hill, Cherry Hill, Curtis Bay, Dunbar-Broadway, Forest Park, Harlem Park, Oldtown, Otterbein, East Baltimore, Pigtown, and Washington Village. [13] The VSP required that individuals live in those respective neighborhoods to place orders but did not restrict based off of any other demographic characteristic. This was done to make sure that customers felt that it was a neighborhood based program and not restricted to individuals meeting certain demographic criteria.

### Data collection

This mixed-method study assessed stakeholder views on the feasibility, sustainability, and efficacy of the VSP through a survey of 93 customers and semi-structured interviews with 14 key programmatic stakeholders (i.e., health department staff, grocers, community partners, customers).

### Survey data collection and analysis

Periodic cross-sectional surveys were conducted through in person and telephone surveys at six VSP hubs that were active in the prior fiscal year (July 1 – June 30 2016). Participants were VSP customers who were at least 18 years old and active participants of the VSP. Exclusion criteria included not having placed at least one order as a customer of the VSP in the past fiscal year, and having participated in the study survey in the past 6 months.

In order to recruit customers, study investigators approached potential participants in-person at the designated VSP hubs during the routine grocery-ordering and grocery pick-up times. Respondents were offered the choice of having the survey read to them and responses recorded by surveyor or self-administering the survey. One open-ended question asked if customers would recommend any changes to the program. The surveys took, on average, 10–15 min to complete and respondents were offered VSP promotional items with a value of no more than $10 for their participation. Survey data were collected until 40% of customers at each site were surveyed. These surveys were determined Non-Human Subjects Research by the Johns Hopkins Bloomberg School of Public Health Institutional Review Board.

Evaluation objectives assessed by the survey included 1) customer satisfaction with the VSP ordering system and staff; 2) impact of the VSP on food buying behavior; 3) barriers to healthy food and beverage access and how the VSP addresses those barriers; and 4) unexpected benefits of the VSP (e.g. building a sense of community) (Table 5 in Appendix 1).

Univariate statistics were used to analyze survey feedback. The open-ended survey item was analyzed by coding similar responses into themes.

### Interview data collection and analysis

Interview data collection covered the first four years of implementation of VSP (2010–2014). In 2015, we conducted 14 semi-structured interviews with key stakeholders who played a role in the development and implementation of the VSP. The BCHD provided the list of stakeholders in the following four categories: health department staff, grocery store affiliates, community partners, marketing/art specialists, and customers. BCHD initially contacted stakeholders to assess their willingness to participate in the interviews. We aimed to have 3–5 participants interviewed within each stakeholder type to represent multiple viewpoints. After initial consent through the BCHD, research staff (JL) independently reached out to each participant by email to obtain consent and then followed up to schedule individual phone interviews. The interviewer (JL) did not have any affiliation with the Virtual Supermarket Program to minimize bias in respondents’ answers. Phone interviews were chosen over in-person interviews due to large distances between the evaluation team and the research participants.

The Principal Investigator (PL) developed an initial semi-structured interview guide based on knowledge from her experience in the development phase of the Virtual Supermarket Program (Table 6 in Appendix 2). Questions were tailored to each stakeholder type. BCHD staff (LF) reviewed the interview guide to ensure that it addressed key domains. The topics covered program logistics and implementation, participant and community adoption, marketing strategies, program sustainability, program impact, and overall successes and barriers of the program. After two pilot interviews, the research team modified the guide to ensure that question stems were tailored to each stakeholder type. The interviewer (JL) was educated about details of the program and trained in conducting semi-structured interviews.

Each interview was recorded, professionally transcribed, and uploaded into Dedoose software (Dedoose Version 5.0.11 Los Angeles, CA: SocioCultural Research Consultants, LLC). Two authors (PL, JL) identified themes using qualitative content analysis. They developed an initial list of deductive codes and then used inductive methods to identify sub-codes within each domain. Both authors (PL, JL) coded transcripts independently and met to refine codes and their definitions. Both coders used a final codebook to analyze all transcripts independently, and resolved discrepancies by consensus. The PI and facilitator reviewed code reports, and met frequently to discuss and finalize the themes based on the reports, and, when warranted, returned to the original data to confirm findings. Themes derived from qualitative content analysis were merged with survey results to develop findings. The stakeholder interview component of the study was also deemed “not IRB regulated” by the University of Michigan Institutional Review Board as it pertains to the quality improvement process and not human subject research.

## Results

The survey (*n* = 93) respondents for whom there is complete demographical data were majority African American, female, and over 60 years of age (Table 1: Characteristics of VSP customer survey respondents)**.**

**Table 1:** Characteristics of VSP customer survey respondents (*n* = 93)

Variables	Mean	Range
Age^a^	70.3	28-93
Number of people living in household^b^	1.2	1–5
Frequency of VSP ordering per month^c^	2.8	1–4
	Frequency	Percent
Gender		
Female	79	85.0%
Race		
African-American	85	92.4%
White	6	6.5%
Asian or Pacific Islander	1	1.1%
Don’t Know	1	1.1%
Prefer not to answer	1	1.1%
Education Level		
8th grade or less	7	7.5%
Some high school	23	24.7%
Graduated from high school/GED	32	34.4%
Some college	22	23.7%
Graduated from college	9	9.7%
Annual household income		
Less than $15,000	34	36.6%
$15,000–24,999	17	18.3%
$25,000–$49,999	2	2.2%
More than $75,000	1	1.1%
Don’t know	5	5.4%
Prefer not to answer	34	36.6%

^a^noting that 85% of the respondents were seniors (>60 years old)

^b^Including the respondent

^c^Ordering occurs once per week at VSP sites

We performed key informant interviews (*n* = 14). Six informants were BCHD staff. These included a community organizer (1); program staff (3); a policy expert (1); and an epidemiologist (1). The other eight stakeholders were community partners (2), grocers (2), Neighborhood Food Advocates (2), customers (3), and marketing staff (1). Two stakeholders answered questions as both customers and neighborhood food advocates.

Four key themes were identified from both the survey and interview questions: 1) VSP addressed transportation barriers and improved food availability; 2) VSP improved food purchasing behavior, created a food justice “brand for the city,” and fostered a sense of community; 3)VSP accepted SNAP benefits in addition to cash and credit; and 4) policy changes are needed to allow processing of SNAP benefits online.

### VSP addressed transportation barriers and improved food availability

The majority of respondents identified the lack of transportation to and from the store as the greatest barrier to food access they had before being a customer of the VSP, followed by the lack of availability of healthy food in the neighborhood, the cost of healthy food, and then the knowledge of how to buy and eat healthy food (Table 2: Survey respondents perceived barriers to food access and how VSP facilitates healthy eating among respondents)**.**

**Table 2:** Survey respondents’ perceived barriers to food access and how VSP facilitates healthy eating among respondents

WITHOUT the Virtual Supermarket Program, these factors made it HARD for me to eat healthy							
	Strongly Agree	Agree	Neutral	Disagree	Strongly Disagree	Don’t know	Prefer not to answer
Availability of healthy food in my neighborhood	42.4%	20.7%	2.2%	31.5%	3.3%	0.00%	0.00%
Transportation to and from the grocery store	39.1%	28.3%	0.00%	26.1%	4.4%	1.09%	1.09%
Cost of healthy food	23.9%	25.0%	3.3%	45.7%	2.2%	0.00%	0.00%
Knowledge of how to buy & eat healthy food	19.6%	25.0%	5.4%	48.9%	0.0%	1.09%	0.00%
WHY does the Virtual Supermarket make it easier to eat healthy?							
						*N*	%
Makes healthy food more available						67	77.9
Means I don’t need transportation to and from the market						56	65.1
Sells affordable healthy food						52	60.5
Helps me know how to buy and eat healthy food						48	55.8

Eighty-six out of 93 survey respondents (92.5%) believed that the Virtual Supermarket likely makes it easier for them to eat healthy. Of these respondents 77.9% felt that this was due to the program making healthy food more available and 65.1% felt that it was because they no longer needed transportation. Over half of respondents attributed an increase in healthy food access to the program selling affordable healthy food (60.5%) and providing knowledge on how to buy and eat healthy food (55.8%).

In-depth interviews also highlighted that eliminating transportation barrier was a key component of convenience needed to promote customer participation.

In March 2010, BCHD chose libraries as the initial sites because library branches served as central safe locations with easy computer access. However, customers found it difficult to carry groceries back from the library.

*“We started by having the program set up in libraries but we quickly learned that if you have trouble getting to the grocery store, you probably also have trouble getting to the library so maybe those aren’t the best sites. So a big lesson learned for us was, yeah, bringing the program to the customer.” –*BCHD staff.

In March 2012, the program piloted their first site in a low-income, senior, public housing facility. By placing the ordering and delivery at the place of residence, customers were more willing to participate, as they no longer had to walk with their groceries.

*“Well, it’s convenient for me because there are times I'm not able to go out and do my own shopping…I am in a wheelchair so, therefore, I have limited access to carrying bags from store to home. It’s convenient because they bring the food here, delivery, and therefore you only have to worry about bringing it up to your apartment once they deliver it.” --*Community Partner and Customer.

*“Being inside of residential areas like low income, senior, disabled housing, or public housing helped us to be a part of an already defined community and it also helps us to be able to have that greater depth there. It means a lot more if your neighbor is the one showing you how to order groceries online for the first time as opposed to somebody from the health department that you don’t know.”* –BCHD Staff.

### VSP improved food purchasing behavior, created a food justice “brand for the city”, and fostered a sense of community

The second domain specifically addressed program efficacy and identified three major outcomes.

First, survey questions specifically asked customers if the VSP had encouraged any change in their food purchasing behavior. Nearly half of survey participants reported that they bought more fruits (47.3%) and vegetables (49.7) and less snacks and deserts (41.9%) since starting to use the Virtual Supermarket Program. However, a large proportion of participants (44.1%) reported buying more juices and sodas as well (Table 3: Customer self-reported change in purchasing behavior)**.**

**Table 3:** Customer self-reported change in purchasing behavior

	I get more	I get the same amount	I get less	Does not apply, I never get this	Don’t know
Fruits*	47.3% 44	38.7% 36	10.8% 10	3.2% 3	0% 0
Vegetables*	49.7% 46	38.7% 36	7.5% 7	3.2% 3	1.1% 1
Meat & Fish*	30.11% 28	38.7% 36	19.4% 18	11.8% 11	0% 0
Dairy	34.4% 32	41.9% 39	11.8% 11	11.8% 11	>0% 0
Juice & Soda	44.1% 41	15.1% 33	15.1% 14	5.4% 5	0% 0
Grains	28% 26	>49.5% 46	12.9% 12	9.7% 9	0% 0
Snacks & Deserts	16.1% 15	31.2% 29	41.9% 39	10.8% 10	0% 0
Toiletries	33.7% 31	44.6% 41	7.6% 7	13.0% 12	1.1% 1

*fresh, frozen, canned

Second, BCHD implementation staff highlighted that the program helped bring positive media attention to the city’s efforts to improve access to healthy foods. BCHD staff agreed that the VSP is an innovative program that took a “luxury” program and “figured out how to make [it] work for a low income audience, both for the community and for the grocer.”

In addition, mass media attention created a food justice “brand” for the city.

*“I think that having this program get spotlighted in the media...actually kind of lifted the profile of Baltimore City as kind of a food justice oriented city. So, even if it’s not just a complete accurate, full-detailed story of the food work in Baltimore, I think that the virtual supermarket actually drew some attention to the city and that probably in the end influenced a lot of the decisions in terms of making that a priority for the city.”*

–BCHD staff.

Third, survey results also highlighted that the program had an unexpected result of fostering a sense of community amongst customers. Overall, the vast majority (79.6%) of respondents felt like being a member of the VSP improved their sense of community. This is likely at least somewhat attributed to the increase in social networks that customers experience. 53 (57%) customers responded that they met new members of their community that they did not previously know because of the program and customers meet about 8 people on average.

### VSP accepted SNAP benefits in addition to cash and credit

The third major theme that emerged was the importance of creating a user-friendly method to accept SNAP benefits in addition to cash and credit payments. Semi-structured interviews with programmatic staff highlighted that successful implementation of the VSP hinged on accepting cash and Supplemental Nutrition Assistance Program (SNAP) benefits as grocery payment methods since the large majority of program participants used an EBT card for SNAP benefits as primary payment and cash for any remaining balance. In addition, many customers did not have credit and/or debit cards, which was the traditional payment method to pay for online groceries. Although customers could place their orders from any computer, federal SNAP regulations required that SNAP electronic banking transaction (EBT) cards be processed at the time of food delivery. A major success for the program was finding a grocer that used a mobile handheld device to accept SNAP benefits through EBT cards at the time of food delivery and pick-up.

*“We were able to do that at our [delivery and pick-up] site, accept the cards, have a handheld machine that was able to swipe and provide the receipt to them, so that was big!” –*BCHD staff.

According to survey respondents, the majority were either “very happy” or “happy” with the ordering process (86.0%) and the grocery pick up process which involved swiping EBT cards at the time of grocery pickup (77.4%; Table 4: Customers’ self-reported satisfaction with the program).

**Table 4:** Customers’ Self-Reported Satisfaction with the program

	Very happy	Happy	Neutral	Unhappy	Very unhappy	Don’t know
Ordering process	43.0% 40	43.0% 40	11.8% 11	0.0% 0	2.2% 2	0.0% 0
Grocery pick-up process	33.3% 39	44.1% 41	19.35% 18	1.1% 1	2.2% 2	**0.0%** 0

In the open-ended survey item, most changes respondents suggested revolved around improving efficiency of the grocery delivery process (i.e. distribution process, wait times, and order mistakes; *n* = 17), food quality and delivery storage methods (i.e. bruised fruit and melted frozen items; *n* = 6), making the ShopRite circular more user friendly (*n* = 5), and increasing discounts (*n* = 4). Interestingly, no customers brought up any difficulties with payment processes which was the major obstacle that programmatic staff discussed in the interviews.

### Policy changes needed to allow processing of SNAP benefits online

Program sustainability was the final major theme addressed in the qualitative semi-structured interviews with programmatic staff. Regarding program maintenance, multiple stakeholders acknowledged that in its current form the VSP may be financially unsustainable. Until July of 2015, the program was 100% foundation funded. Now the City of Baltimore supports roughly 30% of the program budget. Roughly, 80% of grant funding supports staff salaries and the remaining funds subsidize delivery costs and marketing efforts.

BCHD staff highlighted that changes to the payment model through federal policy changes could reduce the need for grant funding to fund BCHD staff to coordinate ordering and pick-up. U.S. Department of Agriculture (USDA) Food and Nutrition Service (FNS) regulations currently only allow SNAP benefits to be processed in person using an electronic banking transaction (EBT) card at the time of grocery pick-up which severely limits the number of online grocers willing to allow SNAP benefits as a payment option. Currently, most online grocers allow customers to pay for their groceries using credit cards while they place the order, minimizing the need for in-person presence during food deliveries.

*“The next phase is what FNS needs to work on is the technology needed to make sure that there is integrity with the SNAP benefits using the EBT (Electronic Banking Transaction) card. It’s an IT issue that they need to address in order for this to be implemented…And, at that point, I would expect that the Virtual Supermarket model will be able to be replicated across the country.” –*BCHD staff.

By allowing customers to use their SNAP benefits online at the time of order placement, similar to the way credits cards are currently used, the grocer could deliver food at any time and it would allow more flexibility for the customers to order and place orders from any location. Consequently, online grocers could expand their payment options to low-income individuals receiving SNAP benefits.

*“I think funding may be less of a need. Once this policy barrier is overcome, then the grocery stores could be really working directly with housing facilities… if it’s run by community members and they can work directly with the grocery stores, grocery stores could take on more and more leadership because it’s just direct sales to them.”*

*-*BCHD staff.

## Discussion

Data gathered from key stakeholders and supporting customer survey data provide important insights into the feasibility, sustainability, and efficacy of the Virtual Supermarket Program pilot. The VSP has played a pivotal role in bringing attention to the issues of food inequities in Baltimore City since the program’s launch in March 2010 and highlighting places for future policy reform. Despite challenges and setbacks, the program has demonstrated that online food stores and food delivery is a feasible, innovative model that may increase access to healthy foods and beverages in low-income underserved neighborhoods.

To our knowledge, only one prior study has evaluated online grocery stores as a model to bring healthy foods to low-income neighborhoods with poor food access. This prior study tested online ordering with a one-time $80 dollar voucher to purchase food from an online grocery store in 34 caregivers in Chicago. [14] They conducted a follow up survey to ascertain participant feedback on barriers and promoters of online food purchasing. Similar to the qualitative findings obtained from programmatic staff, participants noted that allowing use of SNAP benefits and increasing convenience and flexibility by placing orders from homes would promote use of online grocery services. Participants also noted that having costs equal or lower than a brick and mortar grocery store would promote usage [14]. Our customer surveys similarly showed that participants were overall satisfied with the logistics of grocery pick-up and delivery, but did note that they would appreciate more food discounts, more user-friendly circulars, and increased quality of the food being delivered. In addition, through both qualitative interviews and surveys, we found that there were unexpected benefits from the VSP program outside of just promoting healthy food buying behavior. The majority of customers felt that the program fostered a sense of community within their respective ordering sites and program implementation staff noticed that the program positively highlighted the entire cities efforts to focus on food justice issues.

Our study also identified areas for future improvement of the program, as well as areas for future policy and research. First, we found that collaborating with more grocers with online ordering capabilities and EBT handheld machines to process SNAP benefits may increase program adoption and implementation. The USDA Farm Bill 2014 [15] does plan to pilot online transactions of SNAP benefits in limited populations, and future research should evaluate the effect of this technologic change on food purchasing patterns. This change expansion should make it easier to duplicate the VSP model. However, in the meantime, one potential solution to allow more grocers to expand to customers using SNAP benefits and cash would be to accept payment at the time of delivery by increasing the number of hand-held devices used by grocery store delivery personnel. In a similar program to promote SNAP purchases at farmers markets, increasing the number of these handheld devices in every stall increased use of SNAP benefit usage by 38% over a 48-month period [16]. Second, VSP stakeholders felt that the VSP would not be sustainable over time with grant funding alone. A cost-benefit analysis could provide data on future program sustainability through food sales profit. This profit may allow grocers to take on direct ownership of the program. Finally, there were also unintended consequences of the VSP, customers also bought more juice and soda through the program, which is similar to research highlighting that customers tend to buy the majority of their calories including unhealthy foods from supermarkets as opposed to fast-food and convenience stores. [17] These findings highlight the need to not only increase access to supermarkets, but also encourage customers to make healthy choices via education and outreach.

This study has multiple limitations. First, stakeholders that were interviewed were contacted based on a list provided by BCHD and may not have been representative of stakeholders who are no longer involved with the program. Given limited resources, we aimed to gather multiple viewpoints from various stakeholder types, and we only interviewed 14 stakeholders via telephone. The data described represent convergent viewpoints. However, there may alternate observations that we did not capture with the limited sample size. Second, we collected surveys using a convenience sample of current customers and this may be representative of all participants. Therefore, we did not have information on those that may have tried the program once and discontinued usage and those that may not have been interested in filling out the survey. Third, we only evaluated a single pilot in one city. This may not be generalizable to other cities or rural and suburban communities.

## Conclusions

Creating innovative programs to increase healthy food and beverage access in disadvantaged communities to help reduce diet-related diseases is a public health priority for multiple national and local entities. Over the past few years, food delivery models through grocery stores and boxed food delivery systems have multiplied but are largely limited to customers that can pay with cash and credit card. In contrast, the VSP program evaluation highlights that an online grocery infrastructure that accepts SNAP benefits in addition to cash and credit is not only acceptable amongst customers but also feasible as an option to increase access to healthy foods for low-income populations. With future research and policy changes addressing food payment models, it will become even easier to disseminate and implement innovative models such as the Virtual Supermarket Program.

## Appendix 1

**Table 5:** Survey Questions and Indicators

Evaluation Objective	Objective Description	Survey indicator
1. To assess VSP participants’ self-reported perceptions of the VSP.	To what extent are the customers satisfied with the various aspects of the program and what changes might increase customer retention in the program	1.1 How happy customers are with various parts of the VSP 1.2 If they would refer a family member or friend to the VSP 1.3 What reported changes they would like to see to the program
2. To assess VSP participants’ self-reported perceptions of how the VSP has affected their food choices (i.e., their diet-related health behaviors).	To what extent has the customers believe that intervention has provided the necessary education and contributed the appropriate means needed to foster self-reliant healthy diet behavior change	2.1 Changes in the amount of different types of food they buy since they have started the program 2.2 If they ate, learned, or bought a new food as a result of nutrition education programming or healthy food incentives 2.3 If healthy food incentives contribute to diet variety 2.4 Changes in the frequency of visits to different types of food vendors
3. To understand self-reported perceptions of how the VSP has changed its customers’ access to healthy food.	To what extent the customers believe that intervention has addressed the common barriers that the community faces to healthy food access	3.1 The VSP makes it easier to eat healthy 3.2 The relationship between the self-reported factors that made it hard for customers to eat healthy and the factors they believe VSP addresses
4. To understand how the VSP affects its customers’ sense of community.	To what extent the intervention has increased the customers’ social networks and their sense of belonging to their communities	4.1 If being a customer improves sense of community 4.2 If customers have met anyone new through the program

## Appendix 2

**Table 6:** Interview Objectives and Sample Questions

Evaluation Objective	Objective Description	Interview indicators
1. To assess perceptions on barriers for customer participation	What obstacles to customers encounter in finding ordering sites and what are ways the VSP has worked toward promoting the reach of the program	1.2 Were there any barriers for customers getting to a participating site (community partner)? 1.3: Was there ever a sense of push/pull when it came to increasing participants for the program versus serving the communities that had the highest need (BCHD staff)? In your opinion, what parts of the campaign made the most impact with the participants (marketing staff)?
2. To assess stakeholder perceptions about the logistics of grocery orders and placed	To assess the barriers to placing orders and picking up food at various sites and to understand how the VSP has worked towards eliminating these barriers	2.1 What were your thoughts about using a computer to order your groceries (customer)? 2.2 Tell me more about how you paid for your groceries (customer)? 2.3 Was it difficult to accept EBT benefits (BCHD staff)? Did you consider accepting WIC? If so, what were the barriers to accepting WIC (grocer)?
3. To understand key leadership strategies in maintaining the program	To understand what is needed from a leadership and organization standpoint to continue to maintain the program and to start similar programs.	3.1 What improvements could the Health Department do to make this program run more successfully (grocer)? 3.2 How did the health department get buy in from various stakeholders and community when establishing the program (BCHD staff)?
4. To understand how to make the program more sustainable	To get a better understanding at financial and policy barriers that need to be addressed to increase the sustainability of the program	4.1 What do you think needs to be done to make the program more financially stable in the future (BCHD staff)? 4.2 What would you do to help this program continue and be even more successful in the years to come (community partner)?
5. Objective: To understand the impact of the program.	To understand how VSP altered food buying and a sense of community (customers) and also how the program has impacted the community (stakeholders)	5.1 Did you find yourself buying more fruits and vegetables through the virtual supermarket (customer)? 5.2 What do you think this program did for your community? 5.3 Did it build any new skills for your community members (community partners)? 5.4 What are your thoughts on how this program has benefitted participants (BCHD staff)? 5.5 What do you view as the program’s greatest successes (BCHD staff)?

## Abbreviations

BCHD: Baltimore City Health Department
SNAP: Supplemental Nutrition and Assistance Program
USDA: United States Department of Agriculture
VSP: Virtual Supermarket Program
